# Noncoplanar intensity‐modulated radiation therapy for young female patients with mediastinal lymphoma

**DOI:** 10.1120/jacmp.v13i6.3769

**Published:** 2012-11-08

**Authors:** Xinyuan Chen, Dawei Jin, Shulian Wang, Minghui Li, Peng Huang, Jianrong Dai

**Affiliations:** ^1^ Department of Radiation Oncology Cancer Institute & Hospital Chinese Academy of Medical Sciences Beijing China

**Keywords:** noncoplanar, mediastinal lymphoma, IMRT, dosimetry, radiation‐induced second cancer

## Abstract

The purpose of this study is to apply noncoplanar intensity‐modulated radiation therapy (Nonco_IMRT) to young female patients with mediastinal lymphoma. Nonco_IMRT was evaluated through a planning comparison study with coplanar IMRT (Co_IMRT) and conventional anteroposterior and posteroanterior fields (AP–PA) plans. Co_IMRT was performed with five equally spaced beams starting from a gantry angle of 216°. Nonco_IMRT used two noncoplanar beams in the sagittal plane to replace the Co_IMRT beams that directly irradiated the breasts. Nineteen young female patients were enrolled in the retrospective study. Dose coverage of the planning target volume (PTV) and the dose delivered to organs at risk (OARs) were analyzed. For all patients, PTV coverage and heart V30 were similar between the two IMRT techniques (p < 0.05). Compared to Co_IMRT, the mean dose delivered and regions receiving a low radiation dose were significantly reduced for bilateral breasts and lungs in Nonco_IMRT (p < 0.05). Breast V5 and lung V5 were relatively reduced by 21% and 12%, respectively. Compared with the conventional AP–PA plan, Nonco_IMRT had better PTV coverage and OARs sparing, except for being larger in V5 to breast and lung. In IMRT for young female patients with mediastinal lymphoma, using of Nonco_IMRT significantly reduces the radiation dose to the breasts and lungs compared with Co_IMRT, and consequently reduces the risk of breast second cancer and pulmonary toxicity. Besides young female patients, Nonco_IMRT can also benefit other mediastinal lymphoma patients.

PACS number: 87.55.D‐

## I. INTRODUCTION

External beam radiation therapy as a treatment for mediastinal lymphoma has evolved dramatically in recent years. Before the advent of intensity‐modulated radiation therapy (IMRT), an anteroposterior and posteroanterior fields (AP–PA) plan was often used with blocks or multileaf collimator (MLC) to reduce lung dose. Recently, an IMRT technique using coplanar beams for mediastinal lymphoma was reported.^1^, ^2^ Coplanar IMRT (Co_IMRT) can achieve better dose conformation and planning target volume (PTV) coverage than conventional AP–PA plans. Moreover, dose to the heart, coronary arteries, esophagus, and spinal cord are lower in IMRT plans. The only disadvantage is that large volumes of normal tissue, including the lungs and breasts, receive low‐dose radiation.

The overall prognosis of patients with early‐stage Hodgkin's lymphoma (HL) is excellent, with an overall 10‐year survival of more than 75%.^(3)^ In patients with primary mediastinal large B cell lymphoma, three‐year overall survival has reached 87%.^(4)^ With current multimodality treatment, most patients achieve lifelong complete remission, but poor health status and second cancers remain serious late effects of treatment.^5^, ^6^ Female HL survivors are at high risk of radiation induced breast cancer, particularly those treated at a young age.^7^, ^8^ Lung cancer is one of the principal causes of death from second cancer following HL.^9^, ^10^ Moreover, irradiation can be also related to late pulmonary toxicity.^11^, ^12^ It is therefore important to reduce the dose delivered to the lungs and breasts during radiation therapy for lymphoma.

We previously used Co_IMRT with five to nine equally spaced beams to treat patients with mediastinal lymphoma.^2^, ^13^ However, realizing the disadvantages of Co_IMRT, such as increased low‐dose irradiation for breast and lung, starting from 2009, we gradually switched to a noncoplanar IMRT (Nonco_IMRT) technique. In Nonco_IMRT, two noncoplanar beams in the sagittal plane replace the two Co_IMRT beams that directly irradiate the breasts.

The purpose of this study was to apply Nonco_IMRT to young female patients with mediastinal lymphoma, and to evaluate its dosimetric features.

## II. MATERIALS AND METHODS

### A. Patient selection, volume definition, and dose prescription

This is a retrospective study. Nineteen young female patients with mediastinal HL (n=11) or primary mediastinal B cell lymphoma (n=8) who had been treated with Co_IMRT were selected for this study. The mean age was 24 years, with a range of 15–36 years. All patients were immobilized with thermoplastic masks and simulated on a CT simulator (Brilliance Big Bore CT, Philips Healthcare, Andover, MA).

The clinical target volume (CTV) was contoured according to department's clinical protocol that was similar to the guideline of Yahalom and Mauch.^(14)^ A uniform three‐dimensional margin of 0.7 cm, including respiration‐caused tumor motion and setup error, was applied to the CTV to create the PTV. Bilateral lungs, bilateral breasts, heart, and spinal cord were defined as organs at risk (OARs). Body excluding the PTV was defined as normal tissue to quantify the integral dose for each plan.

The prescribed dose for this study was 36 Gy in 18 fractions. At least 95% of the PTV received 100% of the prescribed dose.

### B. treatment planning

For each patient, a conventional AP–PA plan and two static IMRT plans were designed. All treatment plans were created using the ${\text{Pinnacle}}^{3}$ treatment planning system (version 9.0; Philips Medical Systems, Milpitas, CA). The photon beam energy for all plans was 6 MV delivered from Varian 600CD linac equipped with 60 pair‐leaf MLC. Dose grid resolution was 0.3×0.3×0.3 cm.

The AP–PA plan comprised anterior and posterior fields. The fields' shapes were conformed to the PTV using the MLC with a 0.5 cm uniform margin. Wedges were used to improve the dose distribution, if needed. Taking into account that mediastinal tumor masses are mostly anterior and to spare the spinal cord, the beam weight of the AP field was set to greater than 55%, whereas that of the PA field was less than 45%. Field weights and wedge angles were adjusted to achieve the optimal AP–PA plans.

**Figure 1:**
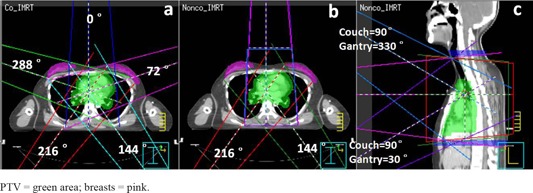
Beam configuration for Co_IMRT and Nonco_IMRT: (a) for Co_IMRT, there were five equally spaced beams (gantry angle 216°, 288°, 0°, 72°, and 144°); (b) for Nonco_IMRT, in the transverse plane, there were three coplanar beams (gantry angle 216°, 0°, and 144°); (c) for Nonco_IMRT, in the sagittal plane, there were two noncoplanar beams (couch angle 90°, collimator angle 90°, gantry angle 330° and 30°). Blocking structures (blue contours) limited noncoplanar beam's irradiation of normal tissue located superiorly and inferiorly outside the PTV.

The beam angles were 216°, 288°, 0°, 72°, and 144° for Co_IMRT plans. Beams were split automatically when the field width was greater than 14.5 cm with a 2 cm overlap. Nonco_IMRT plans also have five beams. Two noncoplanar fields (couch angle 90°, collimator angle 90°, gantry angles 330° and 30°) in the sagittal plane replaced the two fields (gantry angles 288° and 72°) of Co_IMRT in the transverse plane, in order to reduce irradiation of large volumes of the breasts. Figure 1 shows the beam configuration for Nonco_IMRT.

PTV=green area; breasts=pink.

The plans were optimized using the method of direct machine parameter optimization (DMPO).^(15)^ Common plan settings were: minimum monitor units (MU) for each segment, five; minimum segment area, $5\;{\text{cm}}^{2}$; and maximum number of segments, 70. The final dose distributions were calculated by the superposition–convolution method.

The planning goal was to have 95% of PTV received the prescribed dose; the dose uniformity requirement was ‐5% to +7%. For OARs, V20 for bilateral lungs was no more than 30%, heart V30 was no more than 30%, V5 for bilateral breasts was no more than 10%, and the maximum dose delivered to the spinal cord planning organ at risk volume (PRV) with 5 mm margin was no more than 36 Gy. In addition, to limit the irradiation by noncoplanar beams of normal tissue located superiorly and inferiorly outside the PTV, we contoured blocking structures (BSs) at the entry point of the two noncoplanar beams, outside the superior and inferior borders of the PTV. Figure 1 shows the contours of the BSs. A maximum dose of 10 Gy was set to BSs in Nonco_IMRT plan. To make a fair comparison, the same constraint was used in Co_IMRT plan, although it was easier to satisfy.

For each pair of Co_IMRT and Nonco_IMRT plans, the optimization objectives were the same and originated from the Co_IMRT plan. In our center, Co_IMRT plans were designed in two steps. The first step aimed to achieve PTV coverage without violating OAR sparing, as mentioned above. The second step aimed to further spare critical OARs (including breast and lung) as much as possible, without compromising PTV coverage.

### C. Plan comparison

Isodose distributions and dose‐volume histograms (DVHs) were compared between the three planning techniques. Dosimetric parameters related to the doses received by the PTV and OARs were compared quantitatively. For the PTV, the parameters recommended in ICRU Report No. 83 were adopted; these were D98% (dose delivered to 98% of the volume of the PTV), D2% (dose delivered to 2% of the volume of the PTV), mean dose, and dose standard deviation. To assess plan quality with respect to the target dose, a conformity index (CI) and a homogeneity index (HI) were calculated for the PTV in each plan.

CI^(16)^ was defined as follows:

(1) $$CI={\left(TVPV\right)}^{2}/(TV\times PV)$$

where *TVPV* is the portion of the PTV within the prescribed isodose volume, *TV* is the volume of the PTV, and *PV* is the treated volume enclosed by the prescribed isodose surface. Higher values of CI indicate better dose conformity in the PTV. The maximum value of CI is unity, which indicates that the prescribed isodose volume exactly overlaps the PTV.

HI was defined as the difference between the doses covering 5% and 95% of the PTV.^(17)^ The equation is as follows:

(2) HI=D5%D95%

A greater value of HI indicates a greater degree of dose heterogeneity in the PTV.

The OAR dose was evaluated as follows. For bilateral breasts, bilateral lungs, and heart, the chosen parameters were the mean radiation dose and the percentage volume that was irradiated at specific doses (e.g., V5, V10, and V20 for the lungs). For the spinal cord, the parameter was the dose delivered to $1\;{\text{cm}}^{3}$. The mean dose delivered to normal tissue was also assessed.

### D. Statistical analysis

The Wilcoxon matched‐pairs signed‐rank test for nonparametrically distributed data was used to compare the AP–PA and Nonco_IMRT plans, and the Co_IMRT and Nonco_IMRT plans. The threshold for statistical significance was p < 0.05 (two‐tailed). All statistical analyses were performed using SPSS Version 17.0 (SPSS Inc., Chicago, IL).

## III. RESULTS

### A. A representative patient

**Figure 2:**
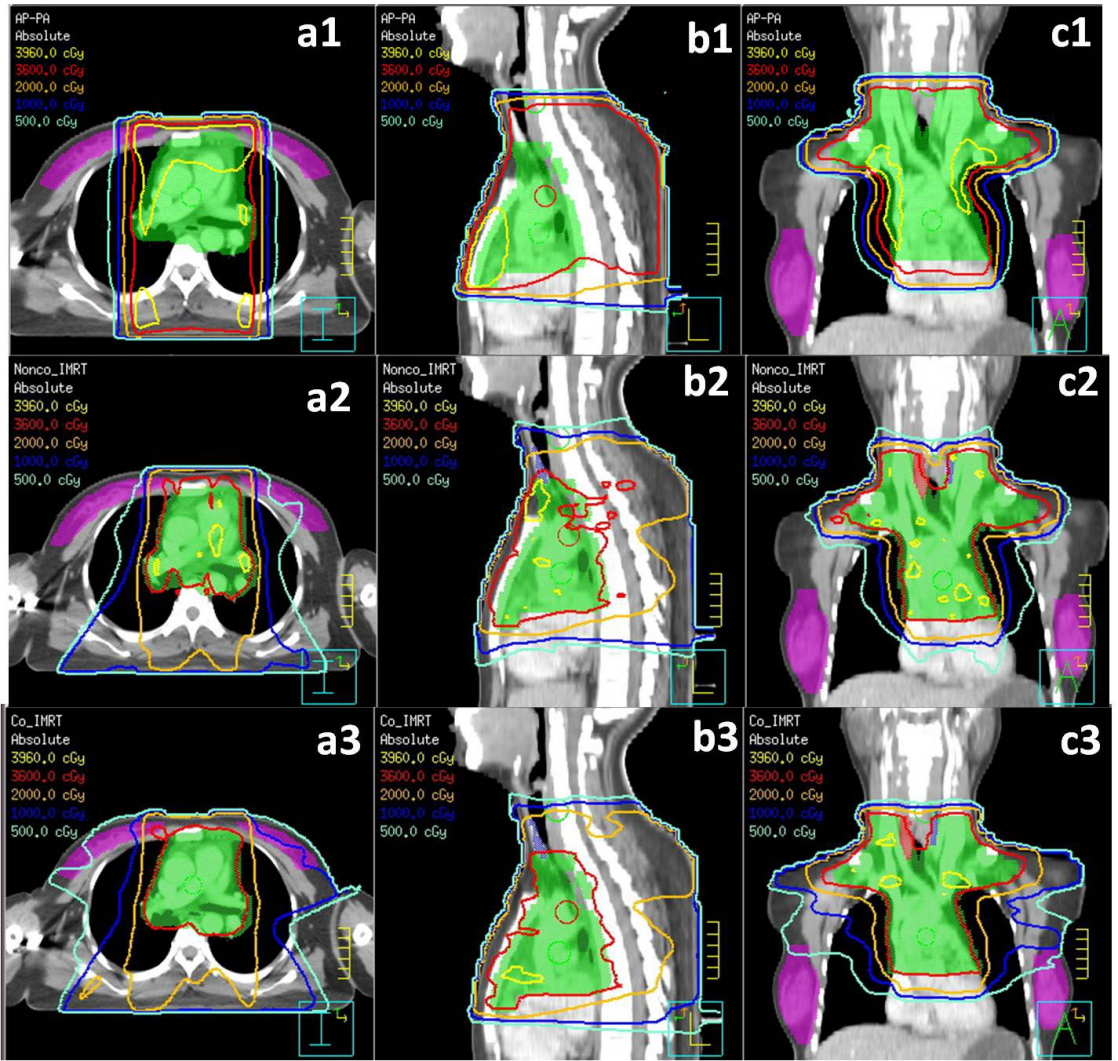
Dose distributions: upper panels show dose distributions for AP–PA treatment in the transverse (a1), sagittal (b1), and coronal (c1) plane; middle panels show dose distributions for Nonco_IMRT in the transverse (a2), sagittal (b2), and coronal (c2) plane; lower panels show dose distributions for Co_IMRT in the transverse (a3), sagittal (b3), and coronal (c3) plane.

**Figure 3:**
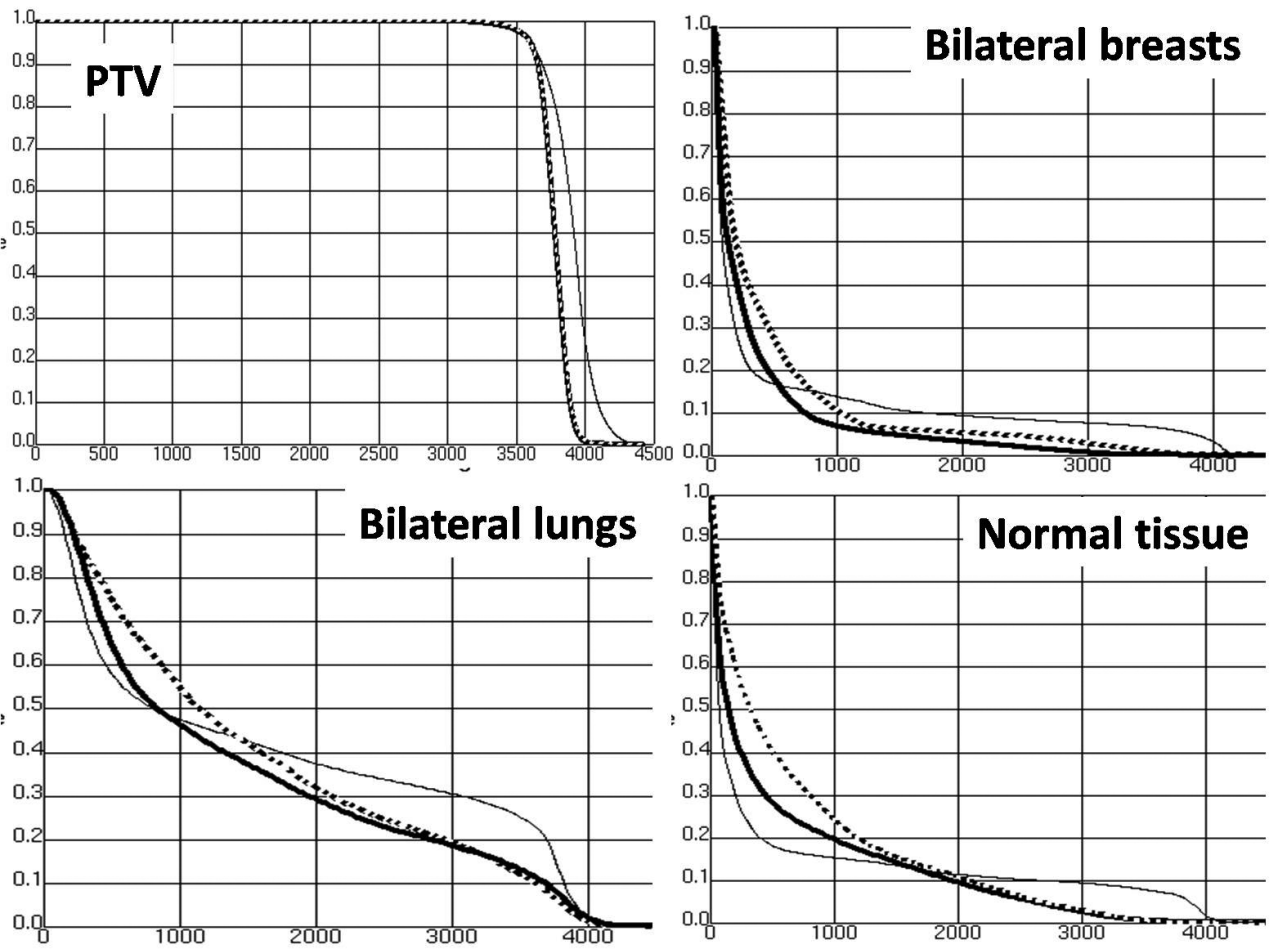
DVH for PTV, bilateral breasts, bilateral lungs, and normal tissue outside the PTV AP–PA plan (thin solid line), Nonco_IMRT (thick solid line), Co_IMRT (thick dashed line).

Figure 2 shows the isodose distribution in the central axial, sagittal, and coronal planes for one representative patient. Figure 3 shows DVHs for the PTV and OARs. It is clearly seen that: a) for PTV coverage, the Nonco_IMRT and Co_IMRT plans were similar, and better than the AP–PA plan; b) for lung and breast, compared with Nonco_IMRT, the 5 Gy isodose line covered greater volumes in the Co_IMRT plan, but smaller volumes in the AP–PA plan; and c) for superior and inferior normal tissue located outside the PTV, the 5 Gy and 10 Gy isodose lines did not show significant increases in Nonco_IMRT compared to Co_IMRT.

The shapes of the DVH curves show that, for control of the delivering relative low doses (e.g., V5) to the lungs, breasts, and normal tissue, Nonco_IMRT was better than Co‐IMRT and worse than AP–PA. For the control of relatively high doses (e.g., V20), Nonco_IMRT was similar to Co_IMRT and better than AP–PA plan.

### B. PtV coverage for all patients

The average PTV volume for the 19 patients was $1311.5\pm 289.0\;{\text{cm}}^{3}$ (range 776.4–$1681.1\;{\text{cm}}^{3}$). PTV coverage for the Co_IMRT and Nonco_IMRT plans was excellent, and approximately equal for all parameters (Table 1). Compared with the AP–PA plan, PTV coverage was much better for Nonco_IMRT, in which PTV D2% and HI were relatively decreased by about 5%, and CI was relatively increased by 51%. Only the average PTV D98% was lower for Nonco_IMRT than for AP–PA, by 0.3 Gy.

**Table 1:** PTV coverage parameters for Nonco_IMRT, Co_IMRT, and AP–PA plans.

				*p‐value*	
	*Nonco IMRT*	*Co IMRT*	*AP–PA*	*AP–PA vs. Nonco_IMRT*	*Co IMRT vs. Nonco IMRT*
D98%(Gy)	34.08±0.82	34.18±0.74	34.38±0.92	0.048	0.075
D2%(Gy)	40.92±0.76	40.89±0.81	43.17±1.25	0.005	0.136
MD^a^ (Gy)	38.32±0.71	38.41±0.68	39.11±1.25	0.017	0.127
CI	0.71±0.038	0.72±0.049	0.47±0.12	<0.001	0.075
HI	1.13±0.027	1.13±0.024	1.19±0.048	0.032	0.209

^a^ MD= mean dose.

### C. OARs doses for all patients

As shown in Table 2, the mean dose, V5 and V10 for the breast were significantly lower in Nonco_IMRT than in Co_IMRT (p < 0.05), with relative reductions of 12%, 19%, and 5%, respectively. Compared with the AP–PA plan, breast V5 was significantly higher for Nonco_IMRT; there was no difference in V10 or mean dose between the two techniques. However, breast V20 for Nonco_IMRT was relatively lower 37% than that for the conventional AP–PA plan.

**Table 2:** Dosimetric parameters for OARs in Nonco_IMRT, Co_IMRT, and AP–PA plans.

					*p‐value*	
		*Nonco IMRT*	*Co IMRT*	*AP–PA*	*AP–PA vs. Nonco_IMRT*	*Co IMRT vs. Nonco IMRT*
Breast	MD^a^ (Gy)	2.86±1.13	3.25±1.38	2.79±1.07	0.063	0.033
	V5(%)	12.98±7.98	15.95±9.66	12.00±9.39	0.027	<0.001
	V10(%)	8.04±4.95	8.43±8.12	8.05±7.01	0.223	0.036
	V20(%)	4.01±3.77	3.97±3.66	6.37±5.12	0.012	0.115
Lung	MD^a^ (Gy)	12.86±1.02	13.79±1.07	13.28±3.79	0.032	0.040
	V5(%)	53.92±8.58	61.22±8.25	50.02±8.95	0.001	0.017
	V10(%)	43.15±6.78	47.95±7.78	42.53±8.01	0.057	0.003
	V20(%)	26.87±2.87	27.92±3.41	32.24±6.48	0.001	0.075
Heart	MD^a^ (Gy)	14.98±5.27	14.51±5.45	17.01±7.17	0.001	0.049
	V30(%)	22.05±11.28	22.39±11.09	34.49±13.01	<0.001	0.107
Spinal Cord	D1cc (Gy)	35.99±1.58	35.51±1.96	41.52±2.37	0.001	0.052
NT	MD^a^ (Gy)	4.76±1.15	5.10±0.96	4.96±1.29	0.046	0.036

^a^ MD= mean dose.

Compared with Co_IMRT, the mean dose, V5 and V10 for the lung were significantly lower in Nonco_IMRT, whereas the difference in V20 was not statistically significant. Lung V5, V10 and mean lung dose, were relatively reduced by 12%, 10% and 7%, respectively. Compared with the AP–PA plan, there was no advantage for Nonco_IMRT in terms of lung V5, but the mean lung dose and V20 were relatively decreased by 3% and 17%, respectively (p < 0.05). There was no difference in V10 between Nonco_IMRT and AP–PA plan.

No significant differences were found between the two IMRT plans for the spinal cord D1cc and heart V30. The mean dose to the heart was 2% higher for Nonco_IMRT (p < 0.05), though the difference was very small in absolute terms and the dose received was far below the tolerance dose. Compared with the AP–PA plan, Nonco_IMRT significantly reduced the maximum dose to the spinal cord, by 13%. Heart mean dose and V30 were relatively reduced by 36% and 12 %, respectively, with Nonco_IMRT (p < 0.05).

The mean dose of radiation delivered to the rest of the body was 7% lower for Nonco_IMRT versus Co_IMRT, and 4% lower for Nonco_IMRT versus AP–PA. Both of these differences were statistically significant (p < 0.05).

## IV. DISCUSSION

Mediastinal tumor masses are irregularly shaped and located in close proximity to critical organs such as lung and breast. High cure rates in mediastinal lymphoma result in long life expectancy; thus, reducing late complications, such as second cancers and cardiac toxicity, are important.^18^, ^19^

### A. Nonco_IMrt vs. AP–PA plans

Comparing Nonco_IMRT with the AP–PA plan, it was difficult to reach an absolute conclusion. Each modality had its own distinct advantages and disadvantages.

PTV coverage was much better in Nonco_IMRT, as reported in other studies.^1^, ^13^

The only advantage of AP–PA plan in lung sparing is lower V5 compared with Nonco_IMRT. It is due to the greater number of beams employed in the latter. And for V10, there was no difference between the two techniques. However, for V20 and mean lung dose, Nonco_IMRT had significant advantages. It is important to note that all these lung parameters have been used as radiation pneumonitis indices.^20^, ^21^ Multiple dosimetric factors that define the shape of the DVH should be considered integrated, rather than a single factor. Indeed, with the use of more conformal techniques for lymphoma patients,^2^, ^13^, ^22^ the acute and long‐term pulmonary toxicities associated with higher V5 and lower V20 should be elucidated in future.

For breast, AP–PA plans were better only for V5, compared with Nonco_IMRT. This finding might be a source of concern when using Nonco_IMRT, because the risk of breast cancer increases with low radiation dose.^23^, ^25^ Breast mean dose and V10 showed no differences between the two modalities. However, breast V20 was significantly lower in Nonco_IMRT. This result could be an advantage for Nonco_IMRT because the risk of second breast cancer increases linearly with radiation dose.^(26)^

Heart mean dose and V30 were significantly reduced for all Nonco_IMRT plans compared with conventional AP–PA plans, which has been blamed for inducing late cardiac complications.^18^, ^19^ Nonco_IMRT was obviously superior for spinal cord sparing, and the mean dose of radiation delivered to normal tissue outside the PTV was 4% lower than in the AP–PA treatment.

In summary, Nonco_IMRT could offer better PTV coverage than AP–PA plan. For OAR sparing, Nonco_IMRT could reduce the dose delivered to spinal cord and heart, lower V20 to breast and lung, and decrease mean dose to normal tissue and lung, except Nonco_IMRT plans irradiated more in V5 to breast and lung than AP‐PA plans.

### B. Nonco_IMRT vs. co_IMRT

Compared with Co_IMRT, the greatest advantage of Nonco_IMRT was the marked reduction of the mean dose, V5, and V10 for the breast. This might help to reduce the risk of secondary breast cancer.^(27)^ Another advantage of Nonco_IMRT was that it significantly reduced the mean dose, V5, and V10 for the lung, while lung V20 was unchanged. This might lead to a lower occurrence of secondary lung cancer and radiation‐induced pulmonary injury.^(28)^

As DVH shown in Fig. 3, compared with Co_IMRT, low‐dose volume control of breast and lung with Nonco_IMRT was closer to that of conventional AP–PA plans. And the noncoplanar technique did not compromise the PTV coverage or OARs sparing compared to Co_IMRT. These results are encouraging and can be explained by the fact that Nonco_IMRT is designed to combine the advantages of Co_IMRT and conventional AP–PA plans. It uses two noncoplanar beams in the sagittal plane to replace the two beams of Co_IMRT in the transverse plane that irradiate the breast directly and penetrate large volume of the lung. For the mediastinal target, Nonco_IMRT retains three beams (gantry angle 216°, 0°, and 144°) in the transverse plane that are the most beneficial for PTV coverage and OAR sparing. As the breasts are located approximately symmetrically, they are irradiated least when the couch angle is set to 90°. The two noncoplanar fields in the sagittal plane have the same impact in terms of OARs sparing as the AP–PA technique; meanwhile, their fluence maps are optimized to further improve PTV coverage and reduce the dose to OARs.

Notably, Nonco_IMRT also has advantage in terms of normal tissue sparing. The mean dose delivered to normal tissue outside the PTV was reduced by 7% compared with Co_IMRT, which might lead to a lower occurrence of secondary cancer.^(27)^ This result was somewhat unexpected. Kan et al.^(29)^ indicated that the use of noncoplanar beams might increase the peripheral dose because of their greater internal scatter, which may increase the area of superior and inferior normal tissue exposed to radiation. In this study, based on our clinical experience, the gantry angle of the noncoplanar beams was set to ±30°, which was not far away from 0°, to avoid delivering extra radiation to the superior and inferior normal tissue and to avoid gantry/couch and gantry/patient collisions. In addition, artificial BSs were delineated just outside the superior and inferior borders of the PTV to control the dose delivered by the noncoplanar beams. These two measures contribute to the greater normal tissue sparing. It must be emphasized that the angles of the noncoplanar beams were the same for all patients in this study. As patients' anatomy and tumor location vary, it would be beneficial to adjust these noncoplanar beam angles to better fit individual patient's anatomy. If the noncoplanar beam with couch angle of 90° and gantry angle of 330° passes through the patient's jaw, the gantry should be adjusted to avoid direct irradiation of jaw, or hyperextended neck setup could be adopted in advance.

Nonco_IMRT does have some disadvantages. One is longer treatment delivery time. Use of two noncoplanar beams necessitates rotating the couch once during each treatment, which takes 1–2 minutes. Another disadvantage of Nonco_IMRT is the slightly higher mean dose delivered to the heart, though another important cardiotoxicity parameter, V30, was similar in the two IMRT techniques. As shown in (Fig. 1c), one of the noncoplanar beams (couch=90°, gantry=330°) spread over a larger heart volume with low‐dose radiation. It may be desirable to place stricter constraints on heart dose in patients with preexisting cardiovascular risk factors.

Given the potential benefit of Nonco_IMRT for breast, lung, and normal tissue, it is reasonable to replace Co_IMRT with Nonco_IMRT for young female patients with mediastinal lymphoma. Moreover, the advantage to the lung and normal tissue of Nonco_IMRT will also benefit other patients with mediastinal lymphoma, including male and older female patients.

## V. CONCLUSIONS

Nonco_IMRT could offer better PTV coverage and OAR sparing than AP–PA plan, except that it could not absolutely finish low‐dose volume control as AP–PA plans. If IMRT is the chosen treatment modality for young female patient with mediastinal lymphoma, our Nonco_IMRT technique significantly reduces radiation dose to the breasts, lungs, and normal tissues, and hence reduces the risk of pulmonary toxicity and of second cancer in the breast and/or elsewhere. Nonco_IMRT can also benefit other patients with mediastinal lymphoma.
